# First clinical diagnosis of FAME3 via commercial Long-Read sequencing reveals mosaic repeat expansion in *MARCHF6 gene*

**DOI:** 10.1007/s10048-025-00835-6

**Published:** 2025-08-11

**Authors:** B. Lakshitha A. Perera, Russell Stewart, Yutaka Furuta, Kimberly M. Ezell, Lynette Rives, Bethany Nunley, Ashley McMinn, Alyson Krokosky, Serena Neumann, Mary E. Koziura, the Undiagnosed Diseases Network, Rizwan Hamid, Joy D. Cogan, Thomas A. Cassini, Eric R. Gamazon, John A. Phillips III, Rory J. Tinker

**Affiliations:** 1 Division of Genetic Medicine, Department of Medicine, Vanderbilt University Medical Center, Nashville, TN USA; 2 Vanderbilt University School of Medicine, Nashville, TN USA; 3 Department of Pediatrics, Division of Medical Genetics and Genomic Medicine, Vanderbilt University Medical Center, Nashville, TN USA

**Keywords:** Repeat expansion disorders, Long-read sequencing, *MARCHF6*, FAME3, Somatic mosaicism

## Abstract

Familial Adult Myoclonic Epilepsy type 3 (FAME3) is a rare autosomal dominant disorder characterized by cortical tremor and epilepsy, caused by a noncoding pentanucleotide repeat expansion (TTTTA/TTTCA)_n_ in the *MARCHF6* gene. Conventional genetic testing often fails to detect this expansion due to its repetitive structure and intronic location. We evaluated a 61-year-old woman with refractory myoclonic and generalized tonic-clonic seizures, whose prior genetic testing—including exome and genome sequencing—was non-diagnostic. Using PacBio HiFi long-read whole-genome sequencing and the tandem repeat genotyping tool TRGT, we identified a pathogenic *MARCHF6* intronic expansion. The proband harbored one allele with 15 *TTTTA* repeats and a second allele with a compound expansion of 661 *TTTTA* and 12 *TTTCA* repeats. Three affected relatives shared similarly expanded alleles, but with increasing repeat size in the latter generations. Importantly, analysis using TRGT-instability revealed repeat mosaicism in all affected individuals, reflected by variability in motif counts across individual sequencing reads. This somatic heterogeneity may contribute to the phenotypic penetrance, variable expressivity and pleiotropism seen in FAME3 disease expression. To our knowledge, this is the first clinical diagnosis of FAME3 using a commercially available long-read sequencing platform, underscoring its diagnostic utility in resolving complex repeat expansion disorders and uncovering biologically relevant mosaicism.

**Supplementary Information:**

The online version contains supplementary material available at 10.1007/s10048-025-00835-6.

## Introduction

Familial Adult Myoclonic Epilepsy type 3 (FAME3) is an autosomal dominant neurological disorder characterized by cortical tremor—primarily affecting the hands and voice—with symptom onset typically between 10-40 years of age. Most affected individuals develop epilepsy, commonly presenting as generalized tonic-clonic seizures, though partial and absence seizures can also occur. While many respond to antiseizure medications, the condition shows variable progression, sometimes leading to cognitive decline. Pathogenicity is attributed to a heterozygous tandem pentanucleotide (TTTTA/TTTCA)_n_ repeat expansion within intron 1 of the *MARCHF6* gene on chromosome 5p15 as outlined in Supplementary Fig. 1 [1–4]. 

Conventional short-read sequencing approaches—including epilepsy gene panels and exome/genome sequencing—often fail to detect these expansions due to their structural complexity and repetitive nature [5, 6]. Emerging evidence suggests that mosaicism—variation in repeat size across different cells—is a common but underappreciated feature of repeat expansion disorders, with important implications for diagnosis and disease expressivity [7–9]. Despite this established genetic cause, several aspects of FAME3 pathogenesis remain unclear. In particular, how these intronic repeat expansions disrupt neuronal function without consistently altering *MARCHF6* mRNA or protein levels is not fully understood [2, 10]. 

Here, we report the application of PacBio HiFi long-read whole-genome sequencing to resolve a longstanding diagnostic odyssey in a 61-year-old woman with adult-onset epilepsy and cortical tremor. Extensive prior genetic evaluations had been non-diagnostic. We demonstrate that long-read sequencing (LRS) successfully identified a pathogenic repeat expansion in *MARCHF6*, confirming the diagnosis of FAME3 and highlighting the diagnostic power of LRS in complex genetic disorders.

## Methods and results

### Medical summary

The proband is a Caucasian female with an unremarkable birth history and normal early development. She experienced her first generalized tonic-clonic seizure at age 38. Multiple electroencephalograms (EEGs) demonstrated generalized epilepsy, with 5 Hz sharp and slow wave discharges most prominent in the parieto-occipital regions.

Her seizures were stimulus-sensitive, triggered by walking on uneven surfaces, exposure to sunlight, or specific visual and auditory stimuli. Episodes often began with subtle eyelid myoclonus and could progress to more generalized jerking, sometimes triggered by routine activities such as phone calls or shopping. Brain MRI (with and without contrast) was normal. Despite treatment with levetiracetam and extended-release valproate, she continued to experience breakthrough myoclonic seizures, particularly on patterned or uneven surfaces. These were typically brief and mild, though she has had two episodes of multifocal myoclonus lasting > 30 min. Prior evaluations—including routine labs, metabolic workup (normal lactate), and repeated imaging—were unrevealing, except for a transient elevation in GGT in 1996. She was referred to the Undiagnosed Diseases Network (UDN) for evaluation in 2016, following a previous non-diagnostic UDN assessment. Long-read genome sequencing was performed in 2024.

### Family history

**Fig. 1:**
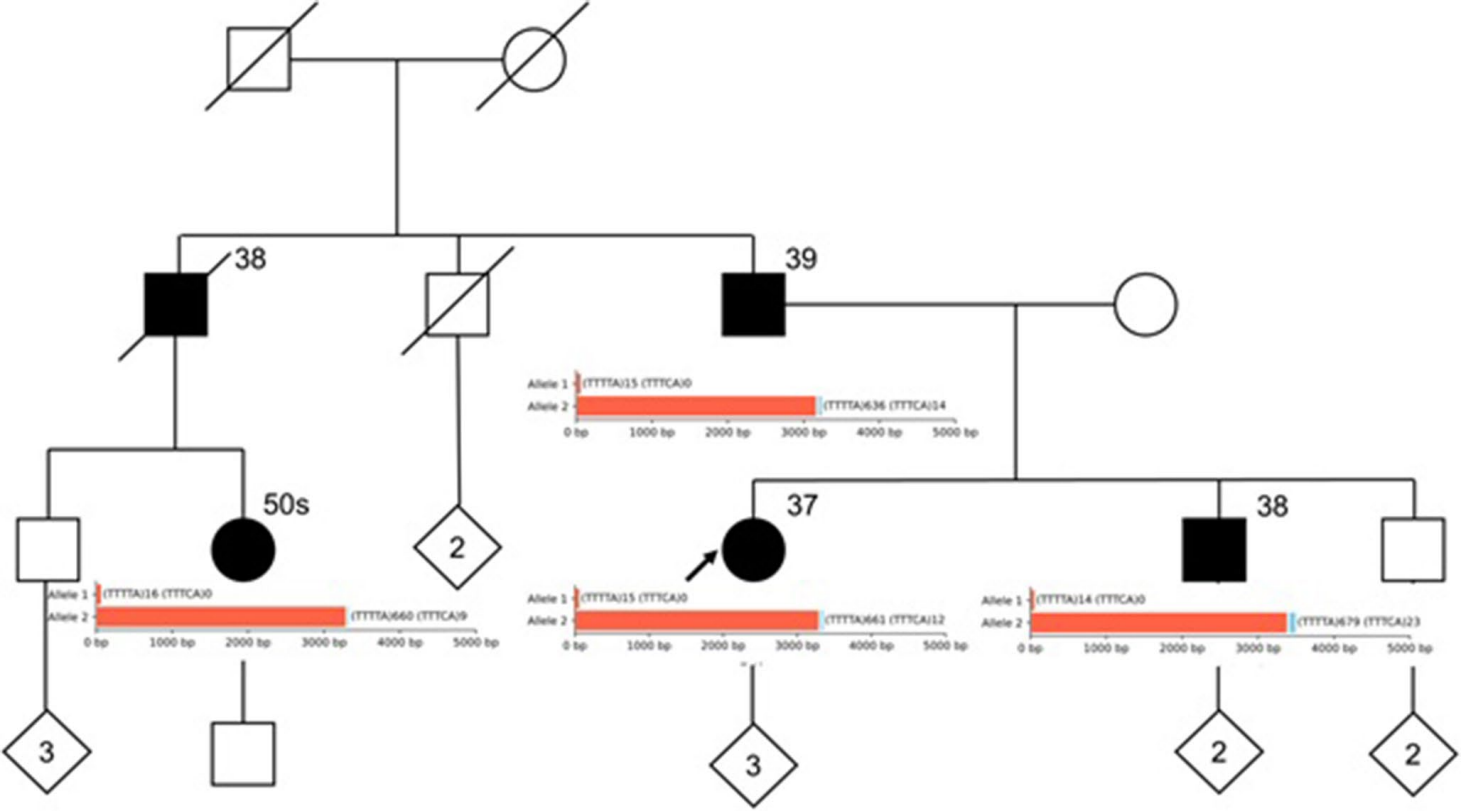
Simplified pedigree overlaid with tandem repeat sequencing data. A limited family pedigree is shown. Tandem repeat counts for (TTTTA)n (red) and (TTTCA)n (blue) for alleles 1 and 2 are superimposed as bar plots. Sequencing data are obtained from PacBio HiFi sequencing data; sequencing depths by allele range from 6 (minimum) to 22 (maximum)

The pedigree demonstrates multiple individuals with adult-onset myoclonic seizures and progressive movement disorders, as outlined in Fig. 1. Affected family members include the proband’s father (onset in the late 30 s), brother (onset in the late 30 s), and paternal female cousin (onset in her 50 s). The paternal uncle, now deceased, reportedly developed symptoms in his late 30s.

## Prior genetic testing

Extensive genetic testing had previously failed to yield a diagnosis. A progressive myoclonic epilepsy panel (sequencing and deletion/duplication analysis) performed at GeneDx was negative. In 2016, an EpiXpanded panel using an exome sequencing backbone was also negative. Genome sequencing performed via the UDN was nondiagnostic.

### Long read sequencing technique

High-molecular-weight genomic DNA was quantified using a Qubit fluorometer and assessed for size distribution with the Agilent TapeStation. Samples achieved Genome Quality Numbers (GQN) ≥ 9.0 at 10 kb and ≥ 5.0 at 30 kb. DNA was sheared into 15–20 kb fragments using Covaris g-tubes, followed by bead cleanup. SMRTbell libraries were prepared using the SMRTbell Prep Kit 3.0 (PacBio), including polymerase binding, primer annealing, and cleanup steps. Libraries were sequenced on the PacBio Revio platform using Revio SMRT Cells, following manufacturer-recommended input concentrations.

### Data analysis

Sequencing data were processed using PacBio’s HiFi-human-WGS-WDL pipeline (v2.1.1) [11]. Reads were aligned to the GRCh38 reference genome, and variant calling was followed by in-house post-processing using custom Python scripts. All bioinformatics analyses were conducted on the Advanced Computing Center for Research and Education (ACCRE) high-performance computing cluster at Vanderbilt University.

## Long read sequencing: Results

Repeat genotyping was performed using the tandem repeat genotyping tool TRGT (v1.5.1) [12, 13] as implemented in the PacBio WGS Variant Pipeline (v2.1.1) [11]. TRGT is optimized for targeted genotyping of tandem repeats from PacBio HiFi sequencing data and generates a consensus sequence per allele using individual reads. This consensus-based approach effectively identifies and reports a single representative repeat structure per allele. However, this summarization can mask underlying variability, particularly in cases of repeat mosaicism, where expanded alleles vary in size across individual reads. To address this, we applied TRGT-instability [14]a recently developed companion tool to TRGT that reports motif composition for each repeat-spanning read. While TRGT outputs a single consensus value per allele—TRGT-instability reveals the true extent of expansion heterogeneity by quantifying motif counts per read, thereby detecting allele-specific instability and mosaicism [12–14]. Using these tools, we focused on six genes previously implicated in FAME: *STARD7*,* YEATS2*,* RAPGEF2*,* MARCHF6*,* SAMD12*, and *TNRC6A* [15]. A pathogenic TTTTA/TTTCA repeat expansion was identified in intron 1 of *MARCHF6* (chr5:10,356,346) in the proband and all three tested affected relatives. The proband had one allele with 15 TTTTA repeats and a second allele with 661 TTTTA followed by 12 TTTCA repeats. Her father, brother, and paternal cousin had similarly expanded alleles, with minor repeat size variations. The repeat structure of the consensus sequences and their allele sizes are summarized in Table 1 and illustrated in supplementary Figs. 2 and 3. No pathogenic repeat expansions were observed in the other candidate FAME genes [15]. 

**Table 1:** Variant and genotypic information for the pathogenic (TTTTA/TTTCA)_n_ repeat expansion observed for *MARCHF6* gene of each individual

Sample	Allele 1				Allele 2				Sequencing Depth	Age of onset
	Length (bp)	TTTTA^*^	TTTCA^*^	Sum of Motifs	Length (bp)	TTTTA^*^	TTTCA^*^	Sum of Motif		
Proband	71	15	0	15	3356	661	12	673	22,7	37
Father	71	15	0	15	3211	636	14	650	15,8	39
Brother	66	14	0	14	3478	679	23	702	6,6	38
Paternal Cousin	76	16	0	16	3340	660	9	669	13,10	50

bp - base pairs; ^*^ The motif count of the consensus sequence spanning the repeat expansion as reported by TRGT

Furthermore, the analysis using TRGT-instability indicated the presence of repeat mosaicism in the expanded allele of all four individuals. The instability of the expanded allele leading to mosaicism has been previously reported in families with MARCHF*6* disease causing variants [5]. The count of each motif observed for individual reads spanning the expansion repeat, and the motif ranges are given in Table 2 and illustrated in the Supplemental Fig. 3.

**Table 2:** The range of repeat motif counts of the individual reads supporting the consensus sequence of the expanded allele (allele 2) as reported by TRGT-instability

Sample	Sequencing Depth	TTTTA^*^	Range	TTTCA^*^	Range
Proband	7	643, 644, 654, 664, 680, 698, 707	643–707	9, 9, 11, 14, 27, 29, 30	9–30
Father	8	603, 609, 613, 639, 642, 642, 651, 659	603–659	9, 10, 11, 11, 14, 18, 22, 91	9–91
Brother	6	619, 678, 679, 680, 683, 701	619–701	9, 10, 23, 27, 46, 110	9-110
Paternal Cousin	10	612, 619, 626, 652, 660, 684, 688, 694, 697, 718	612–718	7, 8, 8, 9, 9, 9, 9, 11, 12, 13	7–13

^*^ The variation of the repeat motif counts between reads in an individual is indicative of repeat mosaicism

### Clinical diagnosis

Based on her clinical phenotype, family history, and LRS findings, the proband was diagnosed with FAME3. Her presentation—adult-onset cortical tremor, stimulus-sensitive myoclonus, and generalized seizures—align with the classical FAME3 phenotype. Identifying a pathogenic intronic repeat expansion in *MARCHF6* in, affected family members, confirmed the molecular diagnosis.

## Discussion

Our study demonstrates the successful use of PacBio HiFi long-read whole genome sequencing to identify a pathogenic TTTTA/TTTCA pentanucleotide repeat expansion in intron 1 of the *MARCHF6* gene, establishing a molecular diagnosis of Familial Adult Myoclonic Epilepsy type 3 (FAME3) in a previously undiagnosed proband and her affected relatives. This is the first report to diagnose FAME3 using a commercially available LRS program. The findings provide compelling genetic evidence supporting autosomal dominant mode of inheritance, with expansion size increasing with one generational transmission.

Our results are consistent with prior reports describing genotype-phenotype relationships in FAME3 [1–4]. As previously shown, the presence of expanded TTTCA motifs, rather than TTTTA repeats alone, appears to be the critical pathogenic element [2, 10]. TTTTA-only expansions are often observed in unaffected individuals and are not considered pathogenic in isolation. Instead, disease seems to arise when TTTCA repeats occur in tandem with TTTTA motifs, suggesting a composite structure that contributes to mRNA-mediated toxicity or genomic instability—rather than altered gene expression or protein dysfunction—as the underlying mechanism of pathogenesis. Our case further supports this model: although the repeat expansions in affected individuals varied in size and composition, all shared the presence of pathogenic TTTCA motifs. This highlights the diagnostic importance of assessing both repeat length and motif composition when evaluating suspected repeat expansion disorders.

Our findings demonstrate that repeat mosaicism is a prominent and likely characteristic feature of *MARCHF6* expansions in FAME3. Using TRGT-instability, we visualized a range of repeat sizes within individual alleles—direct evidence of somatic repeat instability that complements prior reports in other repeat expansion disorders [10]. To our knowledge, this is the first report to directly visualize *MARCHF6* repeat mosaicism at the single-read level in a clinically diagnosed FAME3 family. Our observation of mosaicism raises questions about the threshold effects for pathogenicity, intergenerational instability, and the marked clinical variability often seen among those who are mosaic *MARCHF6* repeat expansion variants.

From a clinical perspective, our findings underscore the transformative potential of LRS in resolving complex or undiagnosed neurological cases. Conventional short-read methods frequently fail to detect non-coding repeat expansions due to alignment and assembly limitations. By contrast, long-read sequencing provides the resolution needed to accurately detect and characterize these complex variants. Incorporating this technology into clinical workflows could substantially improve diagnostic yield, inform more accurate genetic counseling, and enable earlier interventions for affected individuals and at-risk family members.

Future efforts should focus on integrating LRS into routine clinical diagnostics and developing guidelines to identify patients most likely to benefit from this technology. As accessibility and cost-effectiveness improve, LRS may become a frontline tool in the evaluation of rare and undiagnosed genetic conditions.

## Conclusions

This study underscores the diagnostic value of PacBio HiFi long-read whole genome sequencing in resolving complex repeat expansion disorders such as FAME3. In addition to detecting dynamic mutations, this approach further offers distinct advantages in identifying structural rearrangements, somatic mosaicism, and epigenetic modifications—features often missed by conventional short-read sequencing. These expanded capabilities have important implications for patients with unexplained genetic epilepsies and related neurogenetic conditions, where standard testing has been inconclusive. Incorporating long-read sequencing into clinical workflows may improve diagnostic yield, inform prognosis, and guide genetic counseling. Continued investigation into the molecular mechanisms of repeat expansion disorders, and other forms of genomic complexity will be essential for advancing precision medicine and developing targeted therapies to improve patient outcomes.

## Supplementary information

Below is the link to the electronic supplementary material.

Supplementary figure 1 Structure of *MARCHF6* and its tandem repeat. Top: Ideogram of chromosome 5 and its cytogenic bands. Center: MARCH6, with exons highlighted as black bars and its pentanucleotide repeat highlighted as a red bar. Bottom: Detail of wild type (TTTTA)n repeat region. hg38 used as reference for alignment (PNG 32.4 KB)

Supplementary figure 2 Allele plots depicting the reads spanning the repeat expansion regions of a) proband, b) father, c) brother, and d) paternal cousin, aligned to the consensus sequence of the respective allele. The consensus sequences generated by TRGT for each allele are highlighted with a black outline on the top of each allele plot. The individual reads supporting the consensus sequences are aligned below them. The flank regions are colored in green, TTTTA repeat motifs in blue, and TTTCA repeat motifs in purple. Any mismatches, insertions, and deletions are indicated by grey bars, black vertical lines, and horizontal lines, respectively (PNG 299 KB).

High Resolution Image (EPS 385 KB)

Supplementary Figure 3 Waterfall plots with the reads spanning the repeat expansion of a) proband, b) father, c) brother, and d) paternal cousin illustrating the repeat mosaicism. The flank regions are colored in green, TTTTA repeat motifs in blue, and TTTCA repeat motifs in purple. Any mismatches, insertions, and deletions are indicated by grey bars, black vertical lines, and horizontal lines, respectively. Exact repeat motif counts are given in Table 2 (PNG 90.5 KB).

High Resolution Image (EPS 303 KB)

## Data Availability

Detailed clinical and bioinformatics information is available on reasonable request.
