# Supplementary figures and images for: First clinical diagnosis of FAME3 via commercial Long-Read sequencing reveals mosaic repeat expansion in MARCHF6 gene
Source: Neurogenetics. 2025 Aug 11;26(1):61. doi: 10.1007/s10048-025-00835-6 (PMC12339610; doi:10.1007/s10048-025-00835-6)

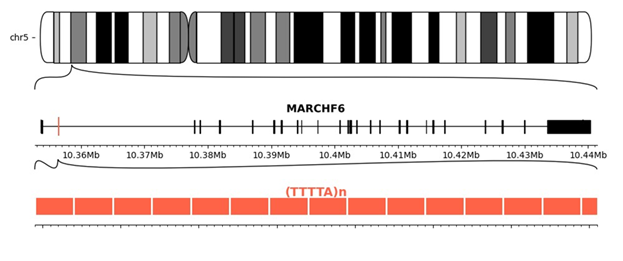

Supplement: Supplementary file 1 — Supplementary figure 1 Structure of MARCHF6 and its tandem repeat. Top: Ideogram of chromosome 5 and its cytogenic bands. Center: MARCH6, with exons highlighted as black bars and its pentanucleotide repeat highlighted as a red bar. Bottom: Detail of wild type (TTTTA)n repeat region. hg38 used as reference for alignment (PNG 32.4 KB) [file 10048_2025_835_MOESM1_ESM.png]

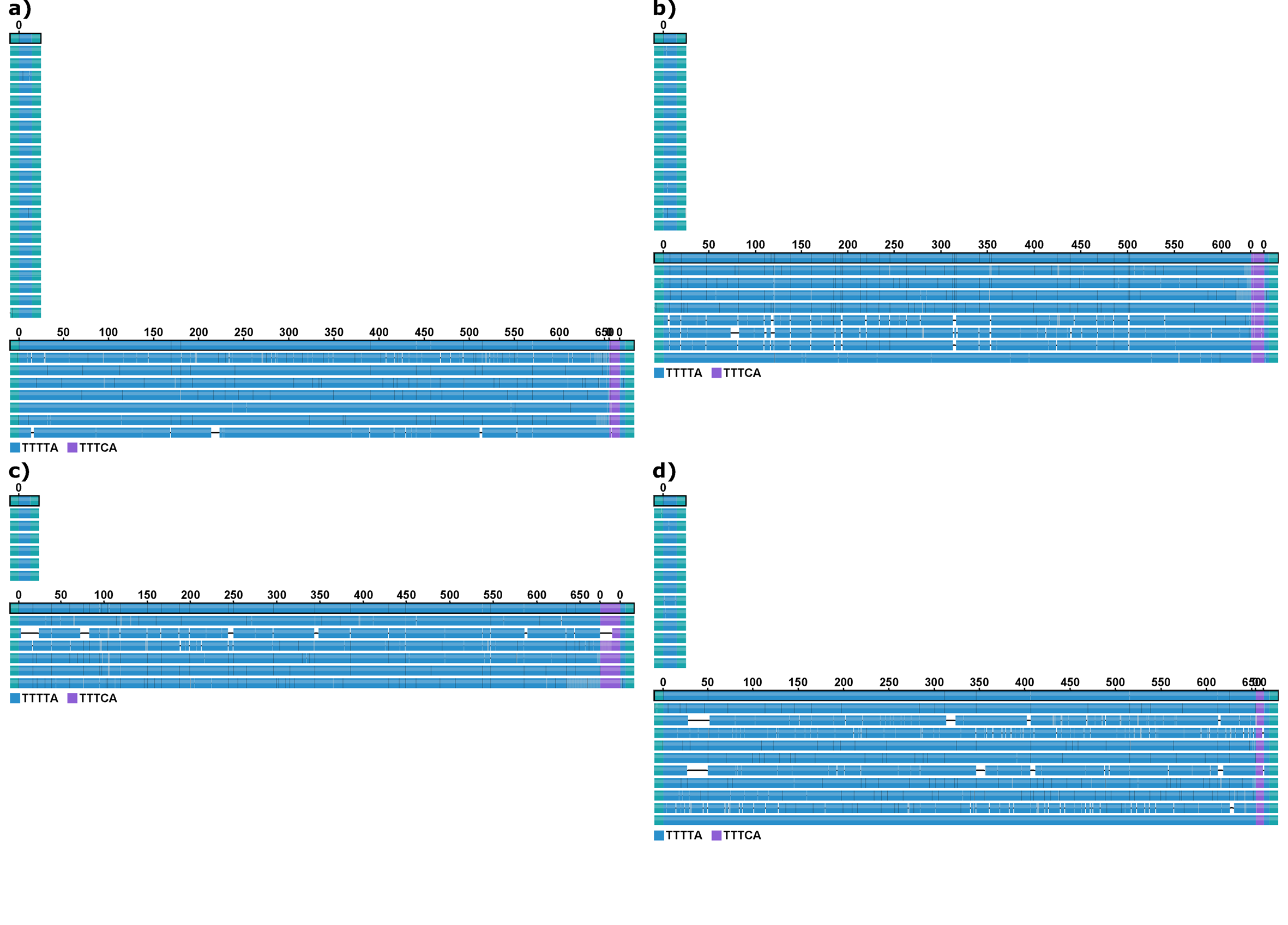

Supplement: Supplementary file 2 — Supplementary figure 2 Allele plots depicting the reads spanning the repeat expansion regions of a) proband, b) father, c) brother, and d) paternal cousin, aligned to the consensus sequence of the respective allele. The consensus sequences generated by TRGT for each allele are highlighted with a black outline on the top of each allele plot. The individual reads supporting the consensus sequences are aligned below them. The flank regions are colored in green, TTTTA repeat motifs in blue, and TTTCA repeat motifs in purple. Any mismatches, insertions, and deletions are indicated by grey bars, black vertical lines, and horizontal lines, respectively (PNG 299 KB). [file 10048_2025_835_Fig2_ESM.png]

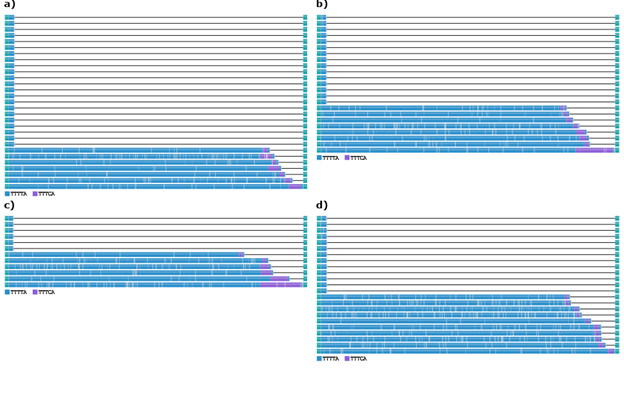

Supplement: Supplementary file 4 — Supplementary Figure 3 Waterfall plots with the reads spanning the repeat expansion of a) proband, b) father, c) brother, and d) paternal cousin illustrating the repeat mosaicism. The flank regions are colored in green, TTTTA repeat motifs in blue, and TTTCA repeat motifs in purple. Any mismatches, insertions, and deletions are indicated by grey bars, black vertical lines, and horizontal lines, respectively. Exact repeat motif counts are given in Table 2 (PNG 90.5 KB). [file 10048_2025_835_Fig3_ESM.png]
